# GammaKnife versus VMAT radiosurgery plan quality for many brain metastases

**DOI:** 10.1002/acm2.12471

**Published:** 2018-10-04

**Authors:** Peter S Potrebko, Andrew Keller, Sean All, Samir Sejpal, Julie Pepe, Kunal Saigal, Shravan Kandula, William F Sensakovic, Ravi Shridhar, Jan Poleszczuk, Matthew Biagioli

**Affiliations:** ^1^ College of Medicine University of Central Florida Orlando FL USA; ^2^ Department of Physics University of Central Florida Orlando FL USA; ^3^ Department of Radiation Oncology Florida Hospital Orlando FL USA; ^4^ Department of Research Florida Hospital Orlando FL USA; ^5^ Nalecz Institute of Biocybernetics and Biomedical Engineering Polish Academy of Sciences Warsaw Poland

**Keywords:** brain radiosurgery, GammaKnife, metastases, VMAT

## Abstract

The purpose of this work was to compare dose distributions between two radiosurgery modalities, single‐isocenter volumetric modulated arc therapy (VMAT), and GammaKnife Perfexion (GK), in the treatment of a large number (≥7) of brain metastases. Twelve patients with 103 brain metastases were analyzed. The median number of targets per patient was 8 (range: 7–14). GK plans were compared to noncoplanar VMAT plans using both 6‐MV flattening filter‐free (FFF) and 10‐MV FFF modes. Parameters analyzed included radiation therapy oncology group conformity index (CI), 12, 6, and 3 Gy isodose volumes (V12 Gy, V6 Gy, V3 Gy), mean and maximum hippocampal dose, and maximum skin dose. There were statistically significant differences in CI (2.5 ± 1.6 vs 1.6 ± 0.8 and 1.7 ± 0.9, *P *< 0.001, *P *< 0.001), V12 Gy (2.8 ± 6.1 cc vs 3.0 ± 5.2 cc and 3.1 ± 5.4 cc, *P *= 0.003, *P *< 0.001), and V3 Gy (323.0 ± 294.8 cc vs, 880.1 ± 369.1 cc and 937.9 ±  vs 361.9 cc, *P *= 0.005, *P *= 0.001) between GK versus both 6‐MV FFF and 10‐MV FFF. No significant differences existed for maximum hippocampal or skin doses. In conclusion, highly optimized VMAT produced improved conformity at the expense of a higher V12 Gy and V3 Gy volume when compared with highly optimized GK.

## 1 INTRODUCTION

The use of stereotactic radiosurgery (SRS) for patients with 1–4 intracranial metastases has been widely accepted as a standard treatment.^1^ A recent study has shown that treating 5–10 brain metastases with SRS is a safe option and results in similar survival compared to patients treated for 2–4 brain metastases.^2^ GammaKnife (GK) radiosurgery has historically been the preferred option.^3^ However, the treatment time for more than five brain metastases with the GK becomes long (1–3 h) especially for aging Co‐60 sources. Furthermore, a long GK treatment ties up valuable clinical resources in a radiation oncology department because of the physical supervision requirement of the radiation oncology physicist and radiation oncologist by regulatory bodies. Linear accelerator‐based SRS is an increasingly utilized alternative to GK because of its wider availability and the potential for rapid (20 min) treatment delivery through high‐intensity flattening filter‐free (FFF) modes.^4^

Liu et al.^5^ demonstrated that plan quality between GK Perfexion and single‐isocenter, multiple noncoplanar VMAT is comparable, with the exception of an increased volume of low dose (<3 Gy) to normal brain using VMAT. However, this study only examined six cases each with 3–4 small brain metastases and only evaluated the dosimetry with 6‐MV FFF plans. Thomas et al.^6^ demonstrated equivalent conformity, dose fall‐off, V12 Gy, and low dose spill between GK and single‐isocenter VMAT using the 10‐MV FFF beam model for 28 cases (median number of targets per case of three). A limitation of their study was the analysis of dosimetry using the older GK Model C and not the GK Perfexion. The GK Perfexion has the potential to provide a dosimetric improvement compared to its predecessor through the convenient delivery of hybrid shots produced by an inverse‐planning algorithm that optimizes target coverage, selectivity, and gradient index.^7^ On the other hand, McDonald et al.^8^ determined that single‐isocenter VMAT delivered significantly more dose to the normal brain compared to GK Perfexion for all dose levels studied. However, their study examined cases with only 2–5 brain metastases and did not use either the 6‐MV FFF or 10‐MV FFF beam models. A recent study by Zhang et al.^9^ looking specifically at hippocampal‐sparing for cases with 3–10 brain metastases (median of six metastases per plan), concluded that GK Perfexion plans demonstrated significantly lower V12 Gy, V8 Gy, and V4 Gy irradiated brain volume compared to single‐isocenter VMAT. However, this study grouped cases with a relatively few number of metastases (3) together with cases containing many metastases (10) and did not compare dosimetry between both the VMAT 6‐MV FFF and 10‐MV FFF beam models.

Our work is the first study that compares GK Perfexion plans to single‐isocenter multiple noncoplanar volumetric modulated arc therapy (VMAT) plans utilizing both VMAT 6‐MV FFF and 10‐MV FFF beam models in patients with seven or more brain metastases.

## 2 METHODS

### 2.A Patients, treatment volumes, and dose

Magnetic resonance (MR) scans of 12 patients with at least seven brain metastases who were originally treated with GK Perfexion were selected for this study. These cases were selected because of long beam‐on times with GK which ranged from 92.3 to 280 min. Gadolinium‐enhanced 2‐mm slice MRI T1‐weighted sequences from a 1.5 T or 3.0 T scanner were used by radiation oncologists and neurosurgeons to contour the gross tumor volume (GTV) in the GammaPlan treatment planning system (v 10.1.1, Elekta AB, Stockholm, Sweden). No margin was added to the GTV to define the planning target volume (PTV). The MRI and structures were exported via DICOM to Eclipse (v 11, Varian Medical Systems, Palo Alto, CA, USA) for VMAT planning. The SRS doses were prescribed according to the size of the lesions following recommendations of radiation therapy oncology group (RTOG) 90‐05^10^ and ranged from 15 to 21 Gy. Doses were modified to respect nearby organ‐at‐risk tolerance, including optic nerve and chiasm maximum doses of 10 Gy and a brainstem maximum dose of 12 Gy.

### 2.B Treatment planning

The GammaPlan treatment planning system was used to generate GK Perfexion plans using the tissue‐maximum ratio (TMR) dose algorithm with a 1‐mm dose grid size and skull measurements.^11^ For each plan, manual shot placement in concert with inverse planning was used to optimize target coverage, selectivity, and gradient index.^7^ Planner adjustments ensured that >99.5% of each lesion was covered by the prescription dose. Out of 103 lesions, the majority were prescribed to the 50% isodose line with 1, 7, 6, 4, and 3 lesions prescribed to the 80%, 70%, 65%, 60%, and 55% isodose lines, respectively. No attempt was made to minimize hippocampal dose.

The Eclipse treatment planning system was used to generate VMAT plans using the progressive resolution optimization algorithm for TrueBeam (Varian Medical Systems, Palo Alto, CA, USA), 6‐MV FFF and 10‐MV FFF beam models with a high definition (2.5‐mm leaf width at isocenter) multileaf collimator (MLC). For consistency, the same homogenous skull volume from GK planning was used for VMAT planning and dose calculation in analogy to the GK TMR dose calculation. The final dose calculation was performed using the Anisotropic Analytical Algorithm using a 1‐mm grid size. All VMAT plans were optimized using 4–6 noncoplanar partial arcs according to the template established by Liu et al.^5^ Dose control tuning structures in the form of volumetric rings were created according to the template established by Clark et al.^4^ to optimize and control dose corresponding to high (prescription dose of each target), medium (12 Gy), and low (6 Gy) dose levels. One isocenter was used for all targets and was placed at the geometric center of mass of all targets calculated by Eclipse. All VMAT plans were generated to ensure that >99.5% of each lesion was covered by the prescription dose. Therefore, a “plan normalization value” between 96.6 and 99.0% was used after VMAT optimization to ensure this target coverage condition. As in GK planning, no attempt was made to minimize hippocampus dose.

### 2.C Dosimetric analysis

The three‐dimensional dose matrices of both GK and VMAT were exported in DICOM RT format to MIM (MIM software Inc., Cleveland, OH) for analysis and comparison. All dose matrices encompassed the entire skull volume at a dose calculation resolution of 1.0 mm. The dosimetric parameters that were analyzed included: RTOG conformity index (CI) and 12 Gy isodose volume (V12 Gy) for each target and patient, as well as 6‐Gy and 3‐Gy isodose volumes (V6 Gy and V 3 Gy), mean/maximum hippocampal dose, maximum skin dose, and beam‐on time for each patient. The RTOG CI = PV/TV, where PV is the prescription dose volume and TV is the target volume.^12^ The VMAT beam‐on time was calculated using a dose rate of 1400 MU/min for 6‐MV FFF and 2400 MU/min for 10‐MV FFF. The GK beam‐on time was recorded from the GK treatment plan printout (GK dose rates of 2.390–3.022 Gy/min).

### 2.D. Statistics

Dosimetric outcomes for GK, VMAT 6‐MV FFF, and 10‐MV FFF plans were compared using a Kruskal–Wallis test because of the non‐normal distribution of values. For statistical analysis of the CI and V12 Gy for each target, each tumor was considered to represent independent information. The V6 Gy, V3 Gy, mean hippocampal dose, maximum hippocampal dose, maximum skin dose, and beam‐on time were tested using patient (N = 12) values with Kruskal–Wallis testing employed. Multiple comparison testing was based on Dunn–Bonferroni tests. SPSS, version 21 was used for analysis with a *P* value less than 0.05 considered statistically significant. No overall correction for multiple testing was applied.

## 3 RESULTS

**Figure 1:**
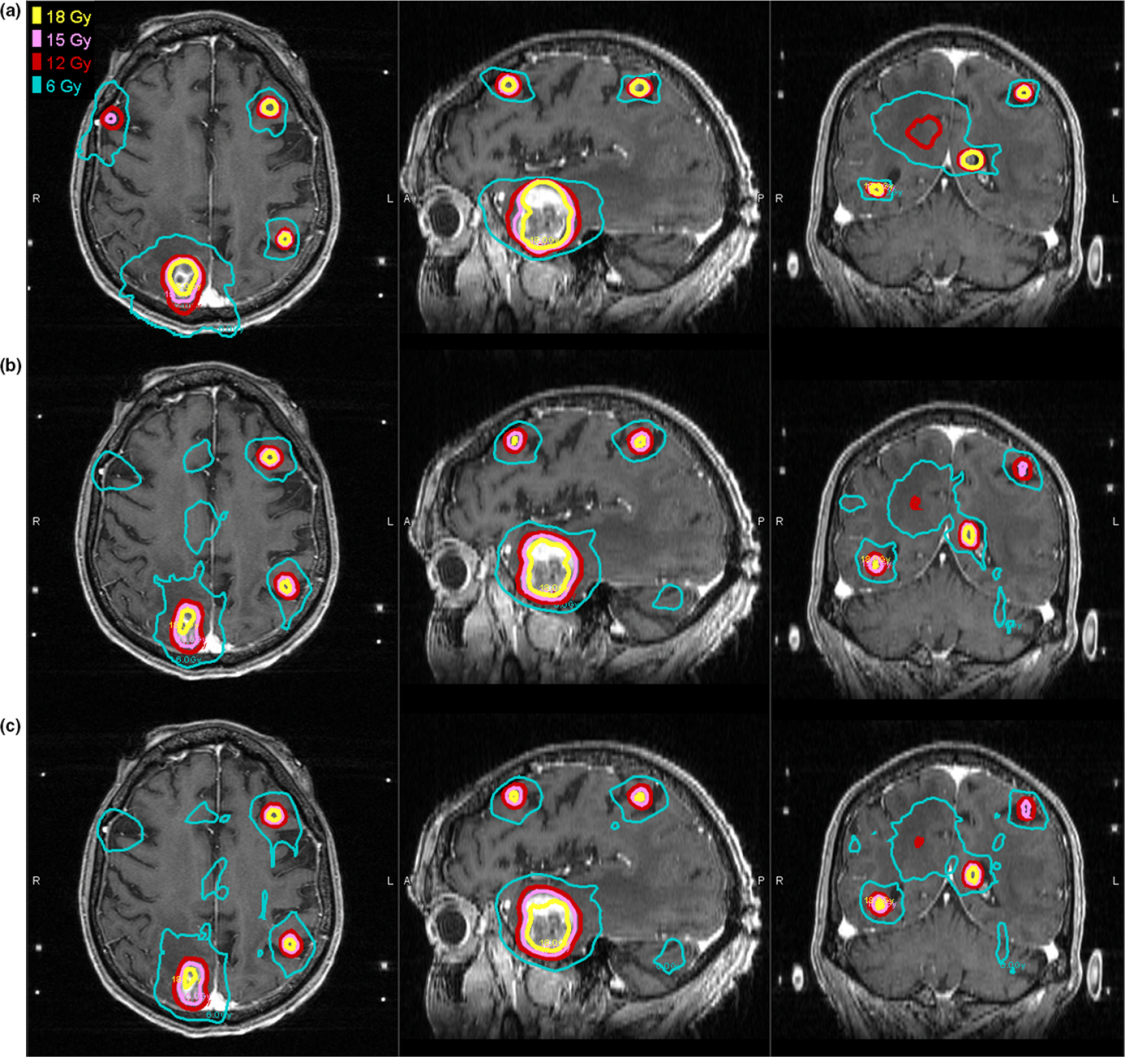
Isodose distributions for (a) GammaKnife (b) VMAT 6 MV FFF (c) VMAT 10 MV FFF plans for a representative patient with 8 brain metastases.

A total of 103 brain metastases were analyzed. Mean tumor volume was 1.16 cc (range: 0.01–19.95 cc) and the median number of targets per patient was 8 (range: 7–14). Figure 1 illustrates the isodose distribution of GK, 6‐MV FFF, and 10‐MV FFF for a typical patient with eight brain metastases. The dose distributions appear qualitatively similar.

Table 1 indicates significantly improved target prescription dose conformity of VMAT compared with GK for both the per‐target (N* *=* *103) and per‐patient (N* *=* *12) analysis. No significant difference in CI between VMAT 6‐MV FFF and 10‐MV FFF was observed. V12 Gy was significantly lower with GK vs both 6‐MV FFF and 10‐MV FFF for the per‐target analysis. However, there was no significant difference in V12 Gy between GK and VMAT for the per‐patient analysis.

**Table 1:** Statistical comparative analysis of plan quality metrics between GammaKnife (GK), VMAT 6 MV FFF, and VMAT 10 MV FFF

Metric	Modality	Mean ± SD	GK vs 6‐MV FFF (p)	GK vs 10‐MV FFF (p)	6‐MV FFF vs 10‐MV FFF (p)
CI (N = 103)	GK	2.5 ± 1.6	<0.001	<0.001	1.0
	6‐MV FFF	1.6 ± 0.8			
	10‐MV FFF	1.7 ± 0.9			
CI (N = 12)	GK	1.6 ± 0.3	0.001	0.001	0.805
	6‐MV FFF	1.2 ± 0.1			
	10‐MV FFF	1.2 ± 0.1			
V12 Gy (N = 103) (cm^3^)	GK	2.8 ± 6.1	0.003	<0.001	1.0
	6‐MV FFF	3.0 ± 5.2			
	10‐MV FFF	3.1 ± 5.4			
V12 Gy (N = 12) (cm^3^)	GK	24 ± 21	0.835	0.705	0.865
	6‐MV FFF	25 ± 17			
	10‐MV FFF	26 ± 18			
V6 Gy (N = 12) (cm^3^)	GK	81.1 ± 72.9	0.09	0.01	1.0
	6‐MV FFF	143.7 ± 81.1			
	10‐MV FFF	167.5 ± 87.5			
V3 Gy (N = 12) (cm^3^)	GK	323.0 ± 294.8	0.005	0.001	1.0
	6‐MV FFF	880.1 ± 369.1			
	10‐MV FFF	937.9 ± 361.9			
Mean Hippo (N = 12) (Gy)	GK	1.9 ± 1.3	0.06	0.01	1.0
	6‐MV FFF	3.4 ± 1.3			
	10‐MV FFF	3.6 ± 1.4			
Max Hippo (N = 12) (Gy)	GK	5.7 ± 6.8	0.1		
	6‐MV FFF	7.1 ± 4.2			
	10‐MV FFF	7.2 ± 4.3			
Max Skin (N = 12) (Gy)	GK	6.9 ± 3.0	0.3		
	6‐MV FFF	5.5 ± 1.8			
	10‐MV FFF	5.4 ± 2.0			
Beam‐on (N = 12) (min)	GK	147.6 ± 49.3	0.01	<0.001	0.02
	6‐MV FFF	10.8 ± 2.1			
	10‐MV FFF	6.4 ± 1.2			

CI, conformity index; Hippo, hippocampus.

The mean tumor volume (1.16 cc) was used as a cutoff value between small lesions (N* *=* *89) and large lesions (N* *=* *14) to determine if there was a difference in GK vs VMAT related to the size of the tumor. Analogous to the results with the total tumor group, both the small lesion group and the large lesion group demonstrated significant improvements in conformity index with VMAT compared to GK (*P *<* *0.001). Also, analogous to the total tumor group, the small lesion group demonstrated a significantly lower V12 Gy for GK compared to VMAT (*P *<* *0.001). However, the large lesion group did not show a significant difference in V12 Gy between GK and VMAT (*P *=* *0.920).

**Figure 2:**
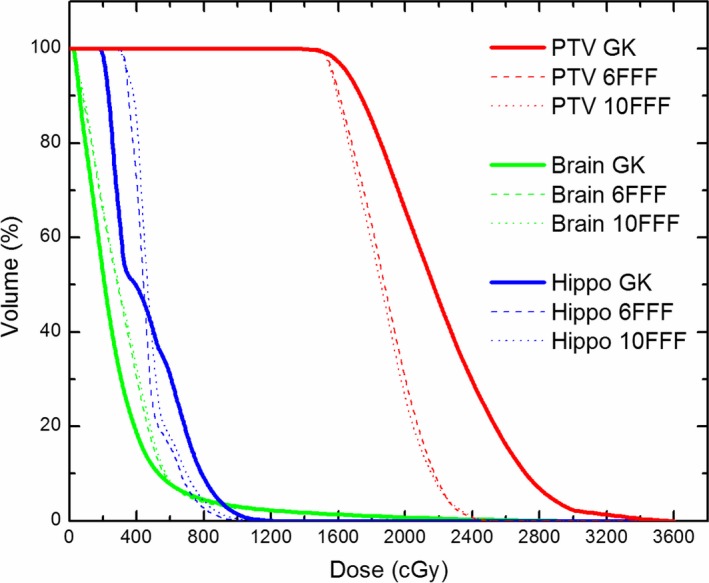
Dose‐volume histograms of the PTV, brain, and hippocampus between GammaKnife (GK), VMAT 6 MV FFF, and VMAT 10 MV FFF plans for the representative patient with 8 metastases. The PTV is the total combined volume of all 8 lesions with prescription doses ranging from 15 to 18 Gy. Notice the larger volume of brain receiving doses less than 6 Gy with VMAT.

For V6 Gy, there was no significant difference between GK and 6‐MV FFF, however, GK was significantly smaller compared to 10‐MV FFF. For V3 Gy, GK was significantly smaller compared to both 6‐MV FFF and 10‐MV FFF. This is illustrated in Fig. 2 which displays the dose–volume histograms for a typical patient, where the PTV is the total combined volume of all eight lesions with prescription doses ranging from 15 to 18 Gy. From Fig. 2, it is apparent that a larger amount of normal brain tissue is irradiated at lower doses (<6 Gy) for the VMAT plans compared to GK.

There was no significant difference between GK and 6‐MV FFF for mean hippocampal dose. However, GK yielded significantly lower mean hippocampal dose compared to 10‐MV FFF. No significant differences existed for the maximum hippocampal and skin doses. GK demonstrated a significantly longer beam‐on time compared to both 6‐MV FFF and 10‐MV FFF.

## 4 DISCUSSION

Our study demonstrated that the volume of brain receiving 12 Gy was statistically significantly smaller with GK than with VMAT for the per‐target (N* *=* *103) analysis. However, this difference was not observed in the per‐patient (N* *=* *12) analysis. A possible explanation for this could be due to the challenge of having different‐sized targets combined into a per‐patient total which adds too much variability while at the same time compressing the sample size, thus making the results nongeneralizable. Liu et al. also analyzed the V12 Gy for each target (N* *=* *19) and patient (N* *=* *6) in their statistical analysis but found no significant difference. Although the clinical implication of the statistically significant difference in our study is unknown, the volume receiving 12 Gy in a single fraction has been demonstrated to predict for both asymptomatic and symptomatic radionecrosis.^13^, ^14^ However, the mean values of V12 Gy for GK and VMAT in our study were well below the threshold of 5.8 cc which has been related to a 10% risk of radionecrosis.^14^ When the mean tumor volume (1.16 cc) was used as a cutoff value between small lesions (N* *=* *89) and large lesions (N* *=* *14), the large lesion group failed to demonstrate a significant difference in V12 Gy between GK and VMAT. This result may be due to the use of the largest (16 mm) collimator in GK to achieve sufficient dose coverage for a large volume and keep treatment times reasonable. However, the sample size (N* *=* *14) is small and a larger study is required to validate this result.

Our results are consistent with the data presented by Zhang et al.^9^ but contradictory to the data presented by Liu et al.^5^ and Thomas et al.^6^ One possible explanation for this may be that our study and the study by Zhang et al. analyzed plans with a median of eight and six targets, respectively per case while the study by Liu et al. and Thomas et al. had only a median of three targets per case. The increased V12 Gy with VMAT for a larger number of lesions may be due to the more complex MLC leaf movement required to simultaneously treat many lesions spread over a geometrically diverse treatment area. It is conceivable that in these scenarios the inverse optimizer must find the global minimum in an increasingly more complex solution space and thus plan quality may degrade.

The major advantage of a linear accelerator‐based treatment using a single‐isocenter to treat multiple targets is the greatly improved efficiency of delivery. However, the drawback of a single‐isocenter treatment is that spatial errors are magnified when the target is not at the isocenter. Therefore, it is common in linear accelerator‐based SRS to add an additional 1–2 mm margin to the GTV for generation of the PTV. This margin accounts for uncertainties in the CT to MR image fusion process, setup error, and the possibility of movement between imaging and treatment with a frameless delivery.^15^ Ezzell^15^ studied the spatial accuracy of two frameless linac‐based SRS systems as a function of distance from the isocenter. The conclusion was that a 1‐mm PTV margin was appropriate for targets up to 7–8 cm from the isocenter while for distances of 10 cm or more, a margin up to 2 mm could be prudent depending on the imaging modality used for alignment. No margin was added to the GTV in our work since we designed our study to use the same volume for VMAT planning that would typically be used in GK planning. However, it should be noted that an additional margin for frameless VMAT delivery would be expected to further increase the V12 Gy compared to GK.

Despite treating a large number of metastases in our study, neither GK nor VMAT violated the hippocampal dose constraints (D100% ≤ 9 Gy and maximum dose ≤16 Gy) recommended by RTOG 0933.^16^ Nevertheless, GK demonstrated a trend toward a statistically lower mean hippocampus dose compared to VMAT (Table 1). This result may be due to the higher photon energies used in linear accelerator SRS and the presence of MLC leakage radiation as discussed below. In our study, no attempt was made to minimize hippocampal dose during VMAT and GK treatment planning. However, the ability to directly take the hippocampus dose into account during VMAT optimization may be advantageous.^17^

Our study also demonstrated a larger volume of brain receiving low doses (≤3 Gy) with VMAT compared to GK. This result is consistent with the findings of Liu et al.^5^ With an increasing number of lesions, VMAT will require large movements of the MLC between scattered targets. This will result in an increase in MLC leakage radiation which may manifest itself as more energy is deposited at low‐dose levels (i.e., ≤3 Gy). This dose level is comparable to a single fraction of whole‐brain irradiation and is considered clinically insignificant. Rahman et al.^18^ analyzed the long‐term risk of secondary malignancy from low doses to normal brain following linac‐based SRS. Based on 23 yr of data, these authors found no increased risk of secondary malignancy compared to the general population.

A limitation of our study was the absence of CT data to produce heterogeneity‐corrected VMAT plans. All of the patients selected for our study were originally treated with GK using MR‐only planning, as is common practice in GK planning, and then replanned with VMAT. We acknowledge that typical VMAT plans would be generated using heterogeneity corrections derived from the electron density information in CT data. However, linear accelerator dosimetry studies using both human skull phantoms^19^ and canine skulls^20^ have found that the impact of heterogeneity correction in the brain, away from low density cavities, was small. Therefore, dose perturbations due to head heterogeneities can be considered a second‐order effect and are not expected to significantly change the results of this study.

With the recent introduction of commercial single‐isocenter planning algorithms for simultaneously treating multiple brain metastases, either using automated dynamic conformal arcs^21^, ^22^, ^23^ or automated VMAT,^24^, ^25^, ^26^ more dosimetric comparison to GK is required. These studies have already demonstrated a reduction in monitor units compared with conventional VMAT. However, dosimetric improvements, if any, need to be better quantified to determine if there is indeed a dosimetric benefit for cases containing a large number of brain metastases. This will be the subject of a future investigation.

## 5 CONCLUSION

Our study is the first work that compares GK Perfexion plans to single‐isocenter multiple noncoplanar VMAT plans and compares both VMAT 6‐MV FFF and 10‐MV FFF beam models for a large number of brain metastases. Although the low‐dose spillage was found to be statistically greater with VMAT than GK, the clinical significance of this remains unknown. For patients requiring a single course of SRS, the improvement in efficiency with VMAT most likely outweighs the small increase in integral dose. However, as many of these patients require multiple courses of SRS, the cumulative effect of low‐dose spillage could become clinically significant.

## CONFLICT OF INTEREST

The authors declare no conflict of interest.

## References

[1] Mehta MP , Tsao MN , Whelan TJ , et al. The American Society for Therapeutic Radiology and Oncology (ASTRO) evidence‐based review of the role of radiosurgery for brain metastases. Int J Radiat Oncol Biol Phys. 2005;63:37–46. 16111570 10.1016/j.ijrobp.2005.05.023

[2] Yamamoto M , Serizawa T , Shuto T , et al. Stereotactic radiosurgery for patients with multiple brain metastases (JLGK0901): a multi‐institutional prospective observational study. Lancet Oncol. 2014;15:387–395. 24621620 10.1016/S1470-2045(14)70061-0

[3] Leksell L . Stereotactic radiosurgery. J Neurol Neurosurg Psychiatry. 1983;46:797–803. 6352865 10.1136/jnnp.46.9.797 PMC1027560

[4] Clark GM , Popple RA , Prendergast BM , et al. Plan quality and treatment planning technique for single isocenter cranial radiosurgery with volumetric modulated arc therapy. Pract Radiat Oncol. 2012;2:306–313. 24674169 10.1016/j.prro.2011.12.003

[5] Liu H , Andrews DW , Evans JJ , et al. Plan quality and treatment efficiency for radiosurgery to multiple brain metastases: non‐coplanar Rapidarc vs Gamma Knife. Front Oncol. 2016;6:26. 26904504 10.3389/fonc.2016.00026 PMC4749694

[6] Thomas EM , Popple RA , Wu X , et al. Comparison of plan quality and delivery time between volumetric arc therapy (RapidArc) and Gamma Knife radiosurgery for multiple cranial metastases. Neurosurgery. 2014;75:409–418. 24871143 10.1227/NEU.0000000000000448 PMC4203364

[7] White Paper: Inverse Planning in Leksell GammaPlan 10. Technical Report Article no. 018880.02, Elekta, 2011.

[8] McDonald D , Schuler J , Takacs I , Peng J , Jenrette J , Vanek K . Comparison of radiation dose spillage from the Gamma Knife Perfexion with that from volumetric modulated arc radiosurgery during treatment of multiple brain metastases in a single fraction. J Neurosurg. 2014;121(Suppl 2):51–59. 25434937 10.3171/2014.7.GKS141358

[9] Zhang I , Antone J , Li J , et al. Hippocampal‐sparing and target volume coverage in treating 3 to 10 brain metastases: a comparison of Gamma Knife, single‐isocenter VMAT, CyberKnife, and TomoTherapy stereotactic radiosurgery. Pract Radiat Oncol. 2017;7:183–189. 28477798 10.1016/j.prro.2017.01.012

[10] Shaw E , Scott C , Souhami L , et al. Single dose radiosurgical treatment of recurrent previously irradiated primary brain tumors and brain metastases: final report of RTOG protocol 90‐05. Int J Radiat Oncol Biol Phys. 2000;47:291–298. 10802351 10.1016/s0360-3016(99)00507-6

[11] White Paper: A new TMR dose algorithm in Leksell GammaPlan. Technical Report Article no. 1021357, Elekta, 2011.

[12] Shaw E , Kline R , Gillin M , et al. Radiation therapy oncology group: radiosurgery quality assurance guidelines. Int J Radiat Oncol Biol Phys. 1993;27:1231–1239. 8262852 10.1016/0360-3016(93)90548-a

[13] Korytko T , Radivoyevitch T , Colussi V , et al. 12 Gy Gamma Knife radiosurgical volume is a predictor for radiation necrosis in non‐AVM intracranial tumors. Int J Radiat Oncol Biol Phys. 2006;64:419–424. 16226848 10.1016/j.ijrobp.2005.07.980

[14] Minniti G , Clarke E , Lanzetta G , et al. Stereotactic radiosurgery for brain metastases: analysis of outcome and risk of brain radio‐necrosis. Radiat Oncol. 2011;6:48. 21575163 10.1186/1748-717X-6-48 PMC3108308

[15] Ezzell GA . The spatial accuracy of two frameless, linear accelerator‐based systems for single‐isocenter, multitarget cranial radiosurgery. J Appl Clin Med Phys. 2017;18:37–43. 10.1002/acm2.12044 PMC5689957 28300379

[16] Gondi V , Pugh SL , Tome WA , et al. Preservation of memory with conformal avoidance of the hippocampal neural stem‐cell compartment during whole‐brain radiotherapy for brain metastases (RTOG 0933): a phase II multi‐institutional trial. J Clin Oncol. 2014;32:3810–3816. 25349290 10.1200/JCO.2014.57.2909 PMC4239303

[17] Birer SR , Olson AC , Adamson J , et al. Hippocampal dose from stereotactic radiosurgery for 4 to 10 brain metastases: risk factors, feasibility of dose reduction via re‐optimization, and patient outcomes. Med Dosim. 2017;42:310–316. 28760560 10.1016/j.meddos.2017.06.007

[18] Rahman M , Neal D , Baruch W , Bova FJ , Frentzen BH , Friedman WA . The risk of malignancy anywhere in the body after linear accelerator (LINAC) stereotactic radiosurgery. Stereotact Funct Neurosurg. 2014;92:323–333. 25277349 10.1159/000365225

[19] Theodorou K , Stathakis S , Lind B , Kappas C . Dosimetric and radiobiological evaluation of dose distribution perturbation due to head heterogeneities for linac and Gamma Knife stereotactic radiotherapy. Acta Oncol. 2008;47:917–927. 17957500 10.1080/02841860701697712

[20] Lyons J , Thrall DE , Pruitt AF . Comparison of isodose distributions in canine brain in heterogeneity‐corrected versus uncorrected treatment plans using 6 MV photons. Vet Radiol Ultrasound. 2007;48:292–296. 17508520 10.1111/j.1740-8261.2007.00245.x

[21] Mori Y , Kaneda N , Hagiwara M , et al. Dosimetric study of automatic brain metastases planning in comparison with conventional multi‐isocenter dynamic conformal arc therapy and Gamma Knife radiosurgery for multiple brain metastases. Cureus 2016;8:e882. 28003946 10.7759/cureus.882 PMC5161262

[22] Narayanasamy G , Stathakis S , Gutierrez AN , et al. A systematic analysis of 2 monoisocentric techniques for the treatment of multiple brain metastases. Technol Cancer Res Treat. 2016;16:639–644. 27612917 10.1177/1533034616666998 PMC5665155

[23] Liu H , Li J , Pappas E , et al. Dosimetric validation for an automatic brain metastases planning software using single‐isocenter dynamic conformal arcs. J Appl Clin Med Phys. 2016;17:142–156. 27685134 10.1120/jacmp.v17i5.6320 PMC5874088

[24] Ohira S , Ueda Y , Akino Y , et al. HyperArc VMAT planning for single and multiple brain metastases stereotactic radiosurgery: a new treatment planning approach. Radiat Oncol. 2018;13:13. 29378610 10.1186/s13014-017-0948-z PMC5789615

[25] Ruggieri R , Naccarato S , Mazzola R , et al. Linac‐based VMAT radiosurgery for multiple brain lesions: comparison between a conventional multi‐isocenter approach and a new dedicated mono‐isocenter technique. Radiat Oncol. 2018;13:38. 29506539 10.1186/s13014-018-0985-2 PMC5836328

[26] Slosarek K , Bekman B , Wendykier J , Grzadziel A , Fogliata A , Cozzi L . *In silico* assessment of the dosimetric quality of a novel, automated radiation treatment planning strategy for linac‐based radiosurgery of multiple brain metastases and a comparison with robotic methods. Radiat Oncol. 2018;13:41. 29544504 10.1186/s13014-018-0997-y PMC5856310

